# Implementation of a Level III neonatal intensive care unit was associated with reduced NICU mortality in a resource limited public tertiary care hospital in Guyana, South America

**DOI:** 10.1371/journal.pgph.0000651

**Published:** 2023-02-15

**Authors:** Sara Singh, Winsome Scott, Caitlin Yeager, Madan Rambaran, Narendra C. Singh, Leif D. Nelin

**Affiliations:** 1 Paediatrics, Georgetown Public Hospital Corporation, Georgetown, Guyana; 2 Institute for Health Sciences Education, Georgetown, Guyana; 3 Guyana Help the Kids, Toronto, ON, Canada; 4 Breen School of Nursing and Health Professions, Ursuline College, Pepper Pike, OH, United States of America; 5 Division of Neonatology, Nationwide Children’s Hospital and The Ohio State University, Columbus, OH, United States of America; PLOS: Public Library of Science, UNITED STATES

## Abstract

Neonatal mortality is a significant contributor to child mortality, and there is increasing interest in low resource settings to implement neonatal intensive care practices to lower neonatal mortality. In Guyana, South America neonatal mortality remains relatively high. At Georgetown Public Hospital Corporation (GPHC), the only tertiary referral hospital in Guyana, a Level III NICU was developed starting in January, 2012 with full implementation in September, 2015. In this study, we report the association of the implementation of a Level III NICU with in-hospital neonatal survival at GPHC. Using an observational study design, available data were collected from January 1, 2015 through September 30, 2020. During the study period, there were 30,733 deliveries at GPHC and 4,467 admissions to the NICU at GPHC. There were no significant changes in the numbers of births or NICU admissions during the time of the study. The survival rate for patients admitted to the NICU was ~64% during the first 3 quarters of 2015 with most deaths were caused by sepsis or respiratory failure. By the last quarter of 2015, the NICU survival rate increased dramatically and has been sustained at ~87% (p<0.0001). The inborn mortality rate at GPHC, calculated as a percentage of all live births at GPHC, was 2.9% prior to the full implementation of the NICU and was 1.4% after the full implementation of the NICU (p<0.0001). These findings suggest that the implementation of a Level III NICU at GPHC was associated with an improvement in survival to NICU discharge in a resource limited setting.

## Introduction

Neonatal mortality is a significant contributor to child mortality (mortality occurring before the age of 5) worldwide, and as measures directed at children 1–4 years of age have had good effect on child mortality, the percentage of child mortality due to neonatal mortality is growing [1]. This underscores the need for interventions directly targeting neonatal mortality. Neonatal mortality is one of the targets for sustainable development goal 3 (3.2), with a target of all countries reducing neonatal mortality to at least as low as 12 per 1,000 live births [1].

The neonatal mortality rate (NMR) remains relatively high in Guyana, South America [1]. The leading causes of neonatal mortality in Guyana are neonatal respiratory distress and neonatal sepsis [2]. Currently, there are a variety of ways that neonatal mortality is being addressed including programs to improve neonatal resuscitation and care, maternal referrals for high-risk pregnancies, regionalization of high-risk pregnancy care, development of tactics to delay or prevent preterm birth, improvement in access to prenatal care, improvements in access to hospital/health center delivery, etc.

As an early part of this more comprehensive program to address neonatal mortality in Guyana, it was decided in January of 2012 to develop a Level III neonatal intensive care unit (NICU) at Georgetown Public Hospital Corporation (GPHC), in apartnership between GPHC, the Ministry of Health (MoH), Guyana Help the Kids (GHTK; a Canadian based NGO), and Nationwide Children’s Hospital (in Columbus, Ohio, USA). Equipment was purchased and training programs in NICU care were instituted for both doctors and nurses. From 2012 through 2015 intensive physician and staff training and equipment procurement led to the full implementation of Level III status for the NICU at GPHC by September of 2015. The fully implemented NICU at GPHC was able to provide mechanical ventilation, nasal continuous positive airway pressure (CPAP), supplemental oxygen therapy, intravenous fluids, surfactant therapy, inotropic medications, diagnostic testing, and pediatric surgery services.

The objective of this observational study was to determine if the implementation of the fully functioning Level III NICU at GPHC was associated with an improvement in NICU mortality prior to hospital discharge. Furthermore, data from UNICEF was used to determine if the implementation of the Level III NICU at GPHC was associated with an improvement in Guyana’s neonatal mortality rate (NMR) over the time of the study.

## Methods

### Ethics statement

This study was approved by the Ethical Review Committee of the Ministry of Health and by GPHC, with a waiver of consent.

### Study design

This was a retrospective single-center observational study using data collected at GPHC starting on January 1, 2015 and continuing through September 30, 2020. Unfortunately, there was no reliable mechanism to collect any kind of consistent NICU data at GPHC prior to January of 2015 due to lack of an electronic medical record and issues with charting. GHTK partnered with the Institute of Health Science Education (IHSE), GPHC, and the University of Guyana to develop a pediatric residency in 2011 at GPHC and as part of this initiative in 2015 a data coordinator was hired to collect various data related to the residency program and neonatal/pediatric outcomes. The fully anonymized data was manually extracted and was limited to data on number of deliveries, admissions to the NICU, and neonatal mortality for each month at GPHC from January 1, 2015 through September 30, 2020. There were a few months in 2017 and 2018 for which data are not available due to lack of personnel to collect the data. The data is available as an Excel spreadsheet in the supplementary material (S1 Data). Data on Guyana’s NMR were taken from the UNICEF database available on-line at https://data.unicef.org.

### Statistics

Data are shown as mean ± SD, number, or rates. Data are graphed versus month and year, or versus year. The categorical data from the time after full NICU implementation is compared to the 3 quarters of 2015 prior to the full NICU implementation using Fisher’s Exact Test (Sigmaplot 14.0, Carlsbad, CA). Linear regression was used to compare 2 variables, for example, NICU survival versus number of NICU admissions (Sigmaplot 14.0). The slopes of NMR versus year for the time period 2006–2015 and 2016–2019 were compared using analysis of covariance (GraphPad Prism 9.0). A p-value of <0.05 is considered significant.

Details of the Development and Implementation of the Level III NICU at GPHC:

1. Space: The first step was to allocate dedicated space apart from other wards in the hospital with availability of multiple sinks throughout the space for good hand hygiene and oxygen and room air piped directly to each bedspace. In 2012 dedicated and separate space was allocated for a NICU at GPHC one floor above labor & delivery. There were multiple sinks available and piped in oxygen at each bedspace, however compressed medical grade air (to provide supplemental oxygen at the desired concentration or for room air CPAP) was only available from tanks placed at the bedside.
2. Equipment: patient monitors, incubators, cots, suction machines, CPAP devices and interfaces, nasal cannula for oxygen delivery, and infusion pumps were obtained and delivered to GPHC by the end of 2012. At the end of 2013 mechanical ventilators were purchased and delivered to the GPHC NICU. By 2015 mechanical ventilation was implemented as evidenced by the ability to intubate appropriate patients, initiate mechanical ventilation, and successfully wean appropriate patients to extubation.
3. Training: A one-year NICU training program for nurses was begun in July of 2012. This one-year training program included 3 months of didactics and 9 months of preceptorship in the NICU. There were regular exams and a comprehensive final exam. Physician training in NICU care and advanced neonatal resuscitation was also started in July of 2012 as part of the pediatric residency program with didactic sessions and rounding with neonatologists either virtually or in-person. An intensive training program for nurses and physicians conducted by visiting respiratory therapists, NICU nurses and neonatologists in how to use the mechanical ventilators was done in early 2014. There were many challenges faced at the beginning and after starting implementation of this program as outlined in the Table 1.

**Table 1:** Challenges in implementation of a Level III NICU at GPHC.

Challenges	Timing	Solutions
*At the beginning of the program*		
Lack of designed, appropriate NICU space	2012	Work with hospital administration to find and outfit appropriate space
Lack of equipment	2012	Purchase and install necessary equipment
No NICU trained staff or physicians	2012	Add NICU skills to pediatric residency curriculum
*After beginning to implement program*		
Thermoregulation—patients getting cold when not in incubators and limited numbers of incubators	2012–2013	Procurement of heating lamps
Implementing CPAP–difficult in providing CPAP using original interfaces	2012–2014	• Procurement of a different interface • Train staff and physician in their use
Lack of skilled NICU nurses–such that specialized equipment/therapies went unused due to lack of training	2012–2015	• Repeat training • Hire Guyanese based RN educator • Work with hospital administration to place NICU trained nurses only in the NICU, rather than rotating throughout the hospital
Implementing mechanical ventilation–difficult to learn when to use, how to wean, and when to extubate	2014–2015	• Intensive re-training • Provision of regular on-going training
Lack of reliable supplemental oxygen	2015	Procurement of reliable oxygen delivery by the hospital

#### Full implementation

By the end of the third quarter of 2015 physician and nurse staffing was reasonable, equipment was functional with all NICU staff well trained in the use of the equipment, and issues with oxygen at the hospital were resolved. Thus, the implementation process took longer than anticipated and there were unforeseen barriers unique to GPHC and Guyana to the implementation of NICU care (see Table 1). Nevertheless, by September of 2015 the NICU was fully functioning as a Level III NICU with coverage by NICU trained nurses and pediatric registrars trained in NICU care and advanced neonatal resuscitation. Although, there continues to be a national nursing shortage, from 2016 on the number of NICU-trained nurses has continued to slowly increase as the one-year certificate programs have continued. The NICU at GPHC is now in new space in a new building connected to labor and delivery, and there are now 4 foreign trained neonatologists working full-time at GPHC.

## Results

**Fig 1:**
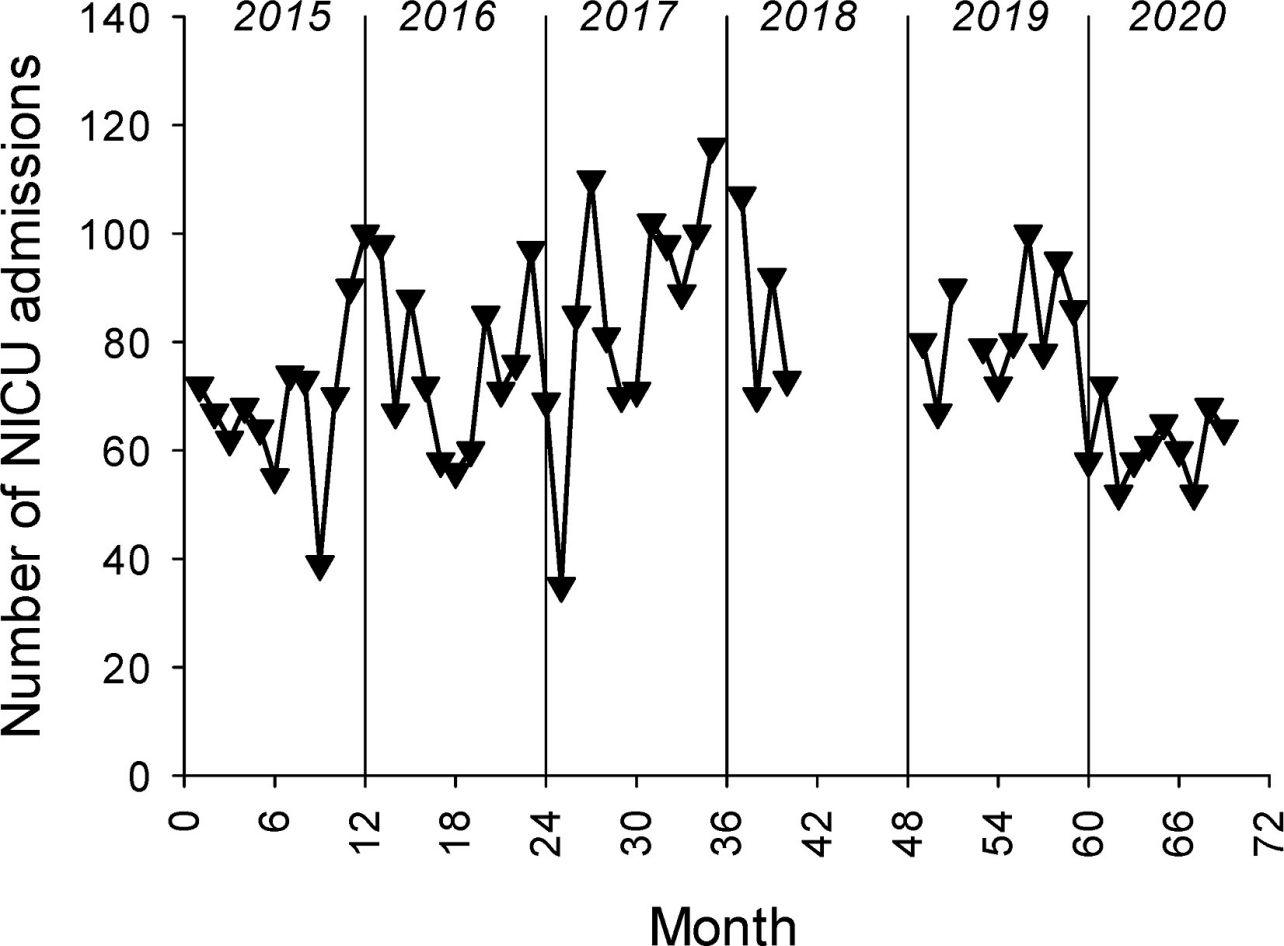
There were slightly more monthly admissions to the NICU following full implementation of NICU care. The monthly number of NICU admissions from January 2015 through September 2020. For the period before NICU implementation (1^st^ 3 quarters of 2015) the mean monthly admissions were 64 ± 11 and for the period following NICU implementation the mean monthly admissions were 77 ± 17, p = 0.03.

**Fig 2:**
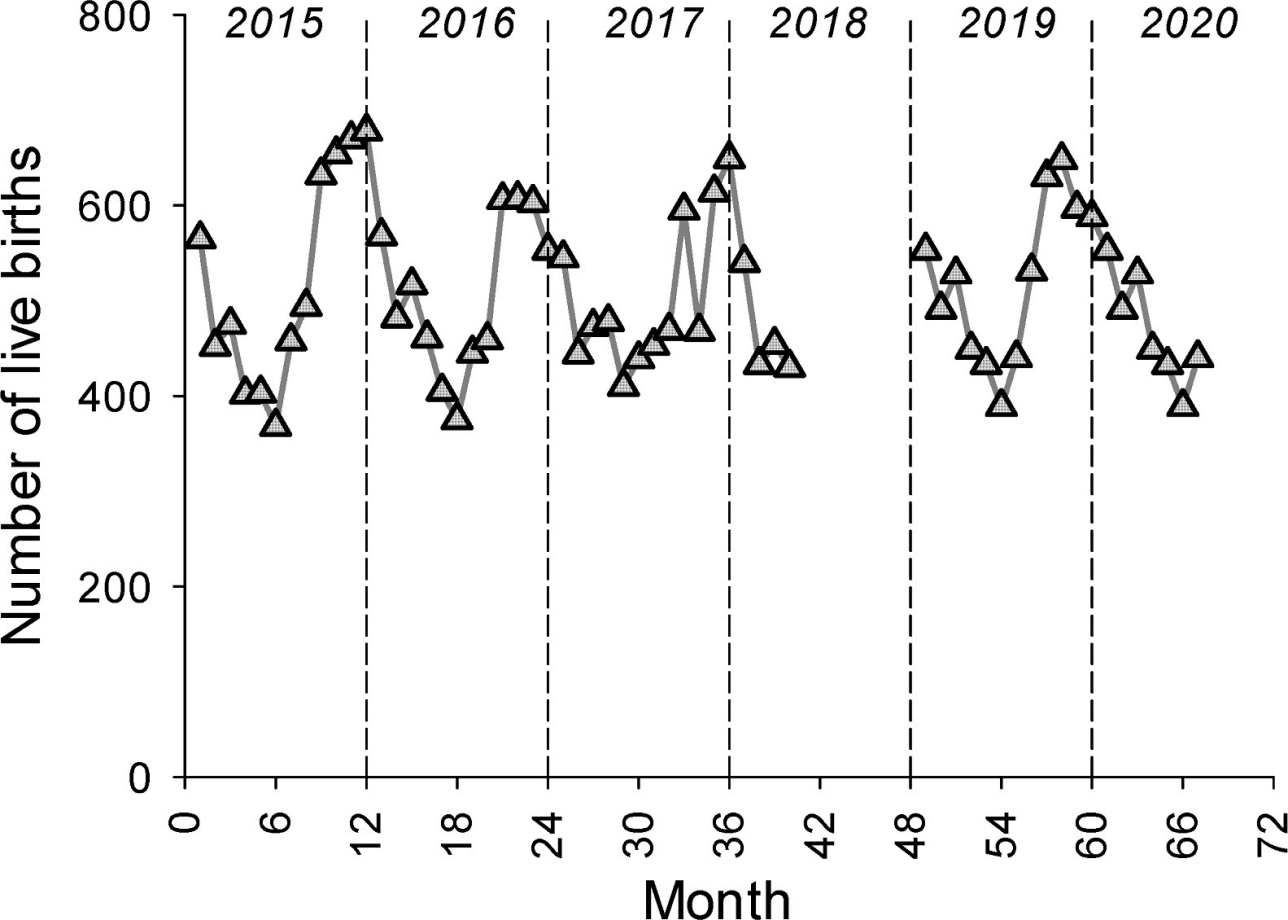
There were no significant changes in the number of live births at GPHC during the study period. The monthly number of live births at GPHC from January 2015 through September 2020. The mean number of monthly live births was 505 ± 84.

There were 4,467 admissions to the NICU at GPHC during the time of the study. As might be expected, there were slightly more monthly NICU admissions after full implementation of the NICU (mean monthly admissions 77 ± 17) than prior to full implementation (mean monthly admissions 64 ± 11, p = 0.03) (Fig 1). There were no substantive changes to NICU admission criteria during the course of the study. The total number of deliveries at GPHC was 30,733 during the time of the study. As shown in Fig 2, although there was a fair amount of month-to-month variability, there were no significant differences in monthly delivery numbers prior to full implementation of the NICU compared to after full implementation of the NICU.

**Fig 3:**
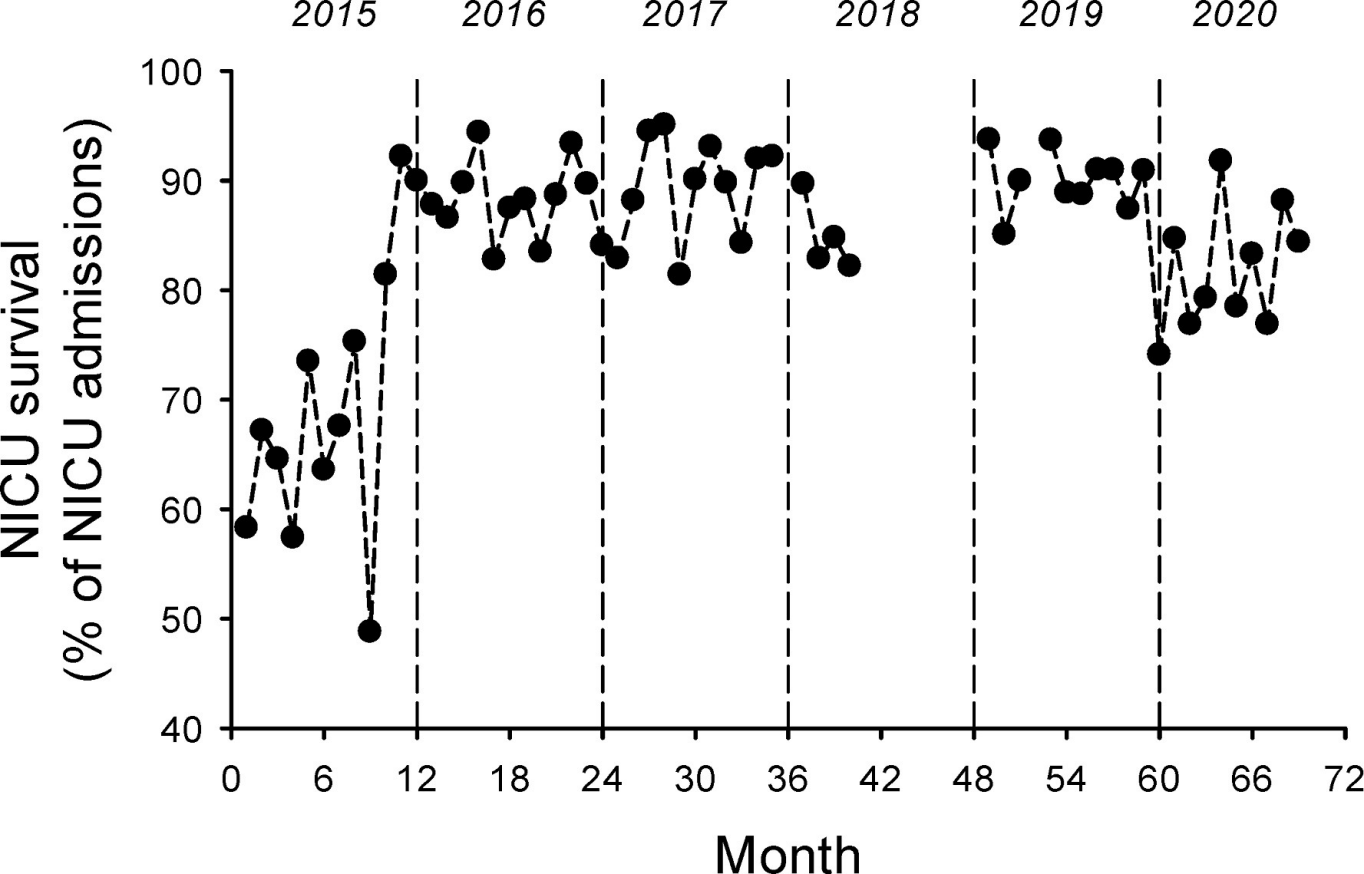
The implementation of the Level III NICU at GPHC was associated with an increase in the monthly survival rate for infants admitted to the NICU. The mean monthly survival rate in the first 3 quarters of 2015 was 64 ± 8% and for 2016–2020 was 87 ± 3% (p < 0.0001), grey dashed lines.

**Fig 4:**
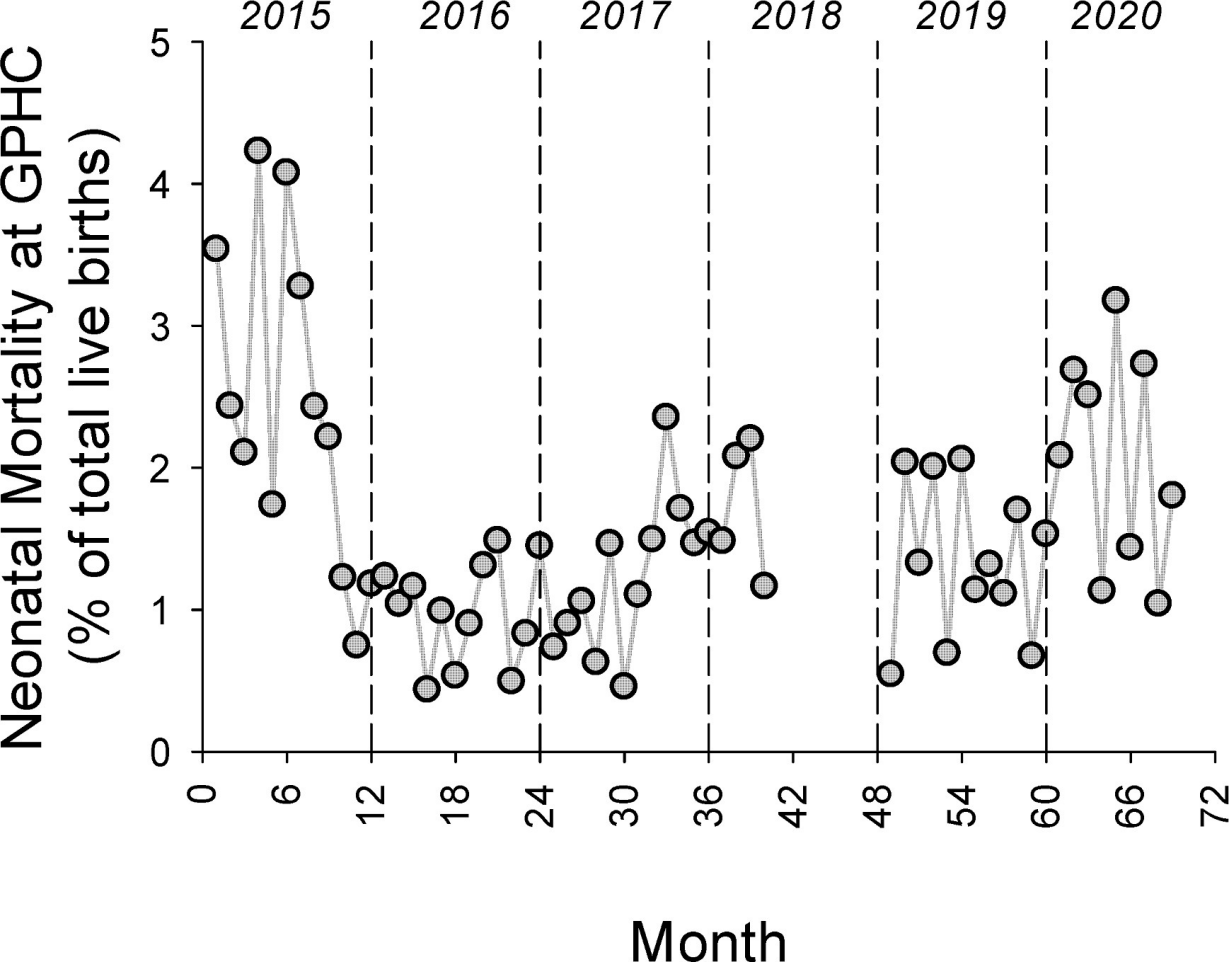
The implementation of the Level III NICU at GPHC was associated with a decrease in the monthly inborn mortality rate as a percent of all live births. The mean inborn mortality rate for all live births at GPHC was 2.9 ± 0.9% for the first 3 quarters of 2015 and 1.4 ± 0.6% for 2016–2020 (p<0.0001), black dashed lines.

The overall survival rate for admissions to the NICU for the entire study period was 84.6% (3,780/4,467). The overall survival rate prior to full implementation of the NICU was 64.8% (372/574) and after full implementation of the NICU the overall survival rate was 87.5% (3,408/3,893), p<0.0001. As shown in Fig 3, the monthly survival rate rose substantially following the full implementation of the NICU and this increase has been sustained. The mean monthly survival rate prior to full implementation of the NICU was 64 ± 8% and after full implementation of the NICU the mean monthly survival rate was 87 ± 5% (p<0.0001). The inborn mortality rate was calculated as a percentage of all live births at GPHC, and Fig 4 shows the monthly inborn mortality rates for the time-period of the study. The overall inborn mortality rate for the entire study period was 1.6% (482/30,733). The inborn mortality rate prior to the full implementation of the NICU was 2.9% and after the full implementation of the NICU was 1.4% (p<0.0001).

Before the full implementation of the NICU at GPHC, infection was the leading cause of death. Surveillance cultures demonstrated mostly Klebsiella before the full implementation of the NICU at GPHC. After the full implementation of the NICU at GPHC, which included strict infection control procedures, the survival rate increased and the leading cause of death changed to respiratory failure. Since the full implementation of the NICU, surveillance procedures have shown that the organisms cultured from NICU patients have changed to include Staphylococcus epidermidis, Staphylococcus aureus, and methicillin resistant Staphylococcus aureus, while pseudomonas and Klebsiella positive cultures are now relatively rare.

**Fig 5:**
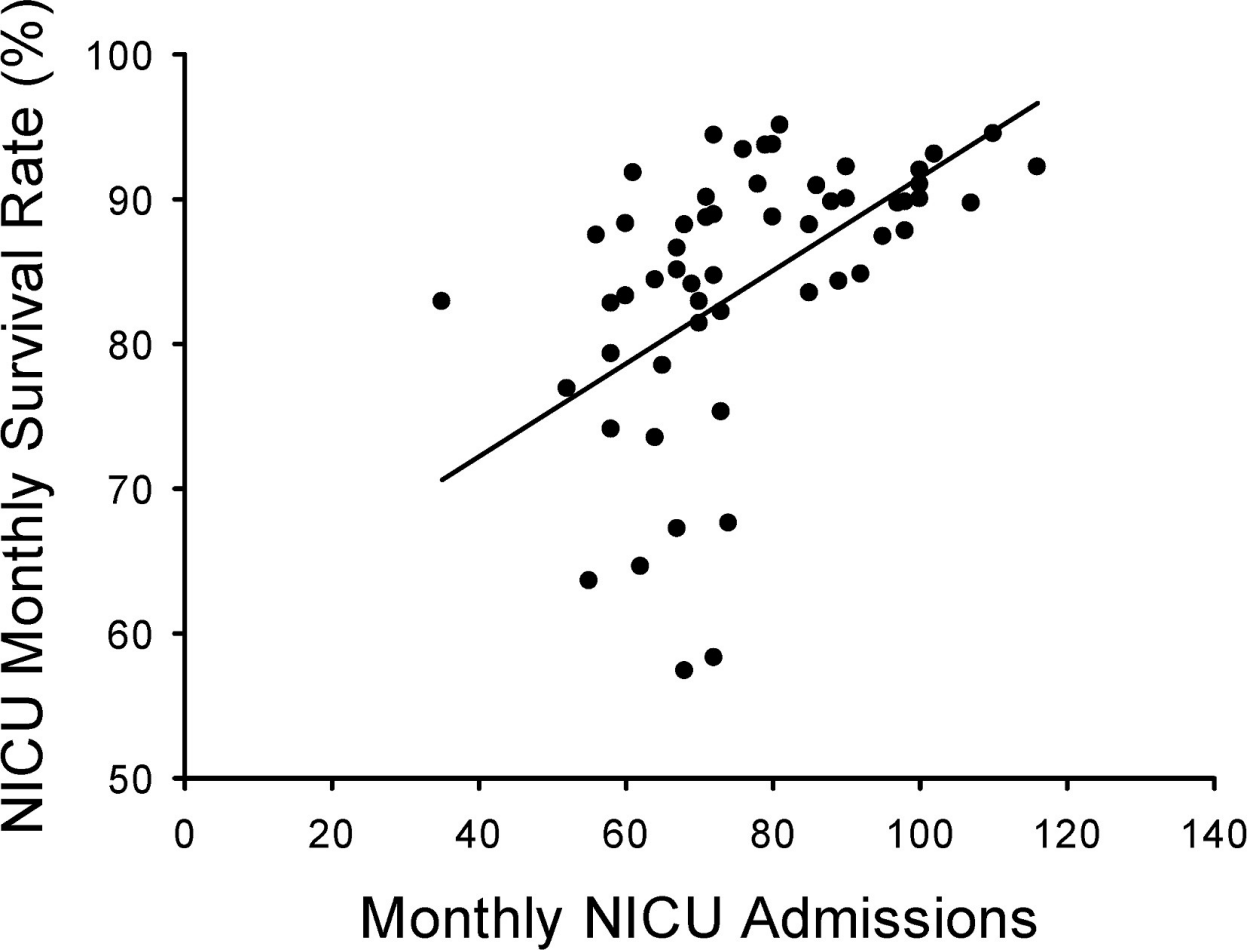
The monthly NICU survival rate was positively correlated with the number of admissions. There was a positive correlation by linear regression analysis between monthly NICU admissions and monthly NICU survival (survival % = (0.32 x number of admits) + 59.4; R = 0.55; R^2^ = 0.30; p<0.001).

Interestingly, we found that in the entire data set the monthly survival in the NICU was associated with the number of admissions in that month by linear regression as shown in Fig 5 (R = 0.55; R^2^ = 0.30; p<0.001). If we used only data from 2016–2020 (i.e., after full implementation of the NICU at GPHC) monthly survival remained associated with the number of monthly admissions by linear regression (R = 0.59; R^2^ = 0.35; p<0.001). These findings suggest a lower mortality rate when there are more admissions to the NICU. There was a weak positive correlation by linear regression between the number of monthly deliveries and number of monthly admissions to the NICU (R = 0.31; R^2^ = 0.10, p = 0.02), while there was no correlation between the total number of monthly deliveries and monthly NICU mortality (R = 0.14; R^2^ = 0.02; p = 0.30).

**Fig 6:**
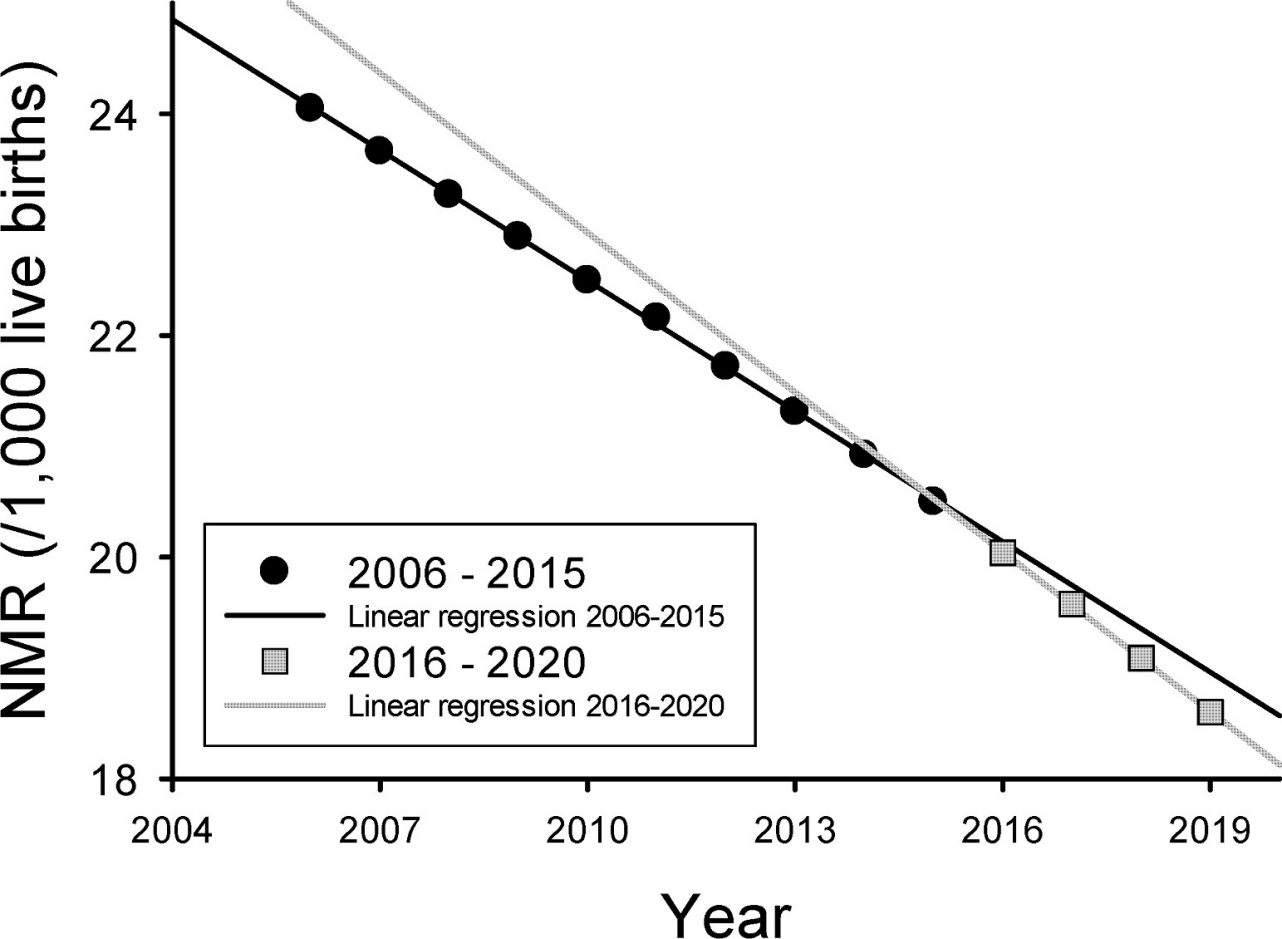
The neonatal mortality rate (NMR) in Guyana, South America from 2006 to 2019 using UNICEF data. The black circles are the NMR from 2006–2015 and the black line is the linear regression fit to the data (y = -0.39x; R = 1.0; p<0.001). The grey circles are the NMR from 2016–2019 and the grey line is the linear regression fit to the data (y = -0.48x; R = 1.0; p<0.001). The slope of the linear regression is the annual rate of change in NMR, a negative slope shows improvement, and the slope for the 2016–2019 NMR was 23% more negative than the slope for the 2006–2015 NMR, and although the differences were relatively small, the two slopes were statistically different, p<0.001.

Finally, to examine if the improvements in NICU mortality rate at GPHC were associated with improvements in the NMR for Guyana we utilized UNICEF data. There was a relatively small and nearly linear annual improvement in the NMR in Guyana from 2006 to 2019 as shown in Fig 6. The rate of annual change in NMR, as given by the slope of the linear regression fit of the data, was –0.39/1000 live births for the period 2006–2015 and increased (was more negative) to –0.48/1000 live births for the period 2016–2020 (p<0.001). This suggests that full implementation of a Level III NICU at GPHC was associated with a slight improvement in the rate of annual change in NMR, but the overall impact on Guyana’s NMR was very small.

## Discussion

The development and full implementation of a Level III NICU at GPHC, the only tertiary care hospital in Guyana was feasible. Although, as is often the case with the development of an entirely new practice paradigm it took a longer time than anticipated for full implementation. To get to full implementation equipment had to be purchased and installed, but perhaps the most important part, and the part that took most time, was training physicians and staff on the use of the equipment so that the equipment could be adopted into routine NICU care practices. By implementing the Level III NICU at GPHC, the diagnoses and conditions that could be treated there increased and that is likely the main reason for the increase in monthly admissions.

In terms of this report, we found that even in a resource limited setting at GPHC the investment, time, and effort required to fully implement a functioning Level III NICU was associated with a sustained increase in NICU survival. Our findings may be generalizable to other resource limited settings. However, when considering development of a new Level III NICU in a setting where there has never been NICU care previously, an approach individualized to the country, setting, and population should be taken as was done at GPHC. This is compatible with reports of the openings of other new NICU in other resource limited settings [3,4]. It should also be pointed out that in high resource settings NICU graduates continue to be at high risk for greater health care utilization, such that a follow-up program is essential, and a follow-up program is currently being instituted at GPHC.

Before the implementation of the Level III status at GPHC, the rate of NICU survival was low at ~65%, and sepsis, particularly Klebsiella sepsis, was a significant contributor to NICU mortality. This is consistent with data from other NICUs in low resource settings [5–9]. Even in a high resource setting Klebsiella can be an important cause of mortality in the NICU and conferred a 3-fold greater risk of mortality than did Staphylococcus aureus [10]. In the GPHC Level III NICU infection prevention resulted in a substantial decrease in Klebsiella infections and a greater percentage of Staphylococcal infections, which are less lethal than Klebsiella and probably underlies at least some of the improvement in NICU survival at GPHC. Sustaining infection prevention at GPHC required strict isolation, strict hand hygiene, visitor restrictions, enhanced cleaning protocols, and repeated reminders/education for the staff and physicians. To maintain lower infection rates requires that a hospital invest in personnel (adequate nursing staffing, house-keeping staff, quality improvement monitoring, etc.) and equipment (functioning incubators, sinks specifically for hand-washing, hand sanitizers/dispensers, data collection, etc.). One key lesson from the implementation of Level III NICU care at GPHC is that infection control, which requires strict attention to detail and continuous meticulous monitoring of the processes necessary to prevent infections, is paramount in improving survival in high-risk neonates.

It has been generally reported that survival in NICUs is greatest in high volume centers even across the same levels of care [11,12]. Interestingly, we found that even in the high-volume center at GPHC, monthly survival rates were positively correlated to monthly admission numbers, such that months with the most admissions also had the highest survival rates (Fig 5). We found this to be somewhat surprising given that high physician and/or staff workloads have been associated with worse NICU outcomes [13]. Unfortunately, we do not have the granular data (such as patient demographics, staffing data, acuity data, etc.) necessary to look for potential associations that might be related to nursing workloads and survival in the GPHC NICU. We can speculate on potential contributing factors to the observed association. First, in months with high numbers of admissions it is possible that more of the admissions are infants with less severe disease (for example, term or near-term babies with transient tachypnea of the newborn, transient hypoglycemia, hyperbilirubinemia, etc.) who have the highest probability of survival. Another potential contributing factor could be that in months with high admission rates more nurses are assigned to the NICU and therefore the nursing provision ratio could potentially be higher than in low admission months (although we doubt that this is a significant contributor to the overall decreased mortality in high admission months because there is a persistent national nursing shortage in Guyana). A third potential contributing factor could be that months with high admission rates actually have low daily census due to admitting infants with lower disease acuity and therefore shorter lengths of stay leading to NICU beds always being available. Such that in low admission months, the NICU may be full of higher acuity patients and therefore NICU beds may not be available for lower acuity infants who are then cared for in the newborn nursery or transferred to other hospitals for care. Although further studies are needed, this association may be an important consideration in the development of new Level III NICUs in resource limited settings, particularly related to design issues, such as numbers of beds and staffing.

We also examined whether the implementation of a Level III NICU at GPHC was associated with a positive impact on the overall NMR in Guyana. The overall NMR in Guyana has been slowly falling at an annual rate of 0.39/1,000 livebirths between 2006 and 2015 (i.e. from 24.05/1,000 live births in 2006 to 20.50/1,000 live births in 2015, see Fig 6). Interestingly, from 2016 to 2019 the annual rate of change in the NMR increased by 23% to 0.48/1,000 live births. Although this improvement in the annual decrease in NMR is encouraging, it must be clearly stated that our study design cannot establish a causal relationship between the Level III NICU at GPHC and the overall NMR in Guyana. Although there was disappointment that the improvements in neonatal survival were not associated with a larger positive effect on the NMR in Guyana, these findings are supportive of the notion that the development of NICUs at tertiary hospitals are not a panacea for improving a nation’s NMR. We postulate that a country specific parallel development of resources that allow large portions of the population to have ready access to higher levels of maternal/neonatal care including transport systems, regionalization of maternal/neonatal care, etc. are also required to positively impact NMR. Regardless, of the cause of the change in NMR unless further improvements to the NMR are made, Guyana may not be able to attain SDG 3.2 by 2030 [1]. It is possible that a large disparity in the NMR between rural regions in Guyana versus the one urban region of Georgetown may be involved, as most births in Guyana happen in the rural regions (MoH data from 2019 shows that 62% of all live births happened in rural regions). The transportation infrastructure for most of the rural regions in Guyana is relatively poor, such that timely transfer of high-risk pregnant women or sick neonates can be very difficult. Thus, it may be that a significant proportion of high-risk deliveries occur in rural areas which lack the necessary expertise and experience in resuscitation and care of high-risk neonates. We have recently begun a project to develop a national neonatal network in Guyana that includes increasing the level of neonatal care in seven rural regional hospitals from Level I to Level II.

This study has limitations that need to be considered. For example, although there were no significant changes in the number of deliveries at GPHC or the number of admissions to the NICU at GPHC, granular data were not available to determine if there were potential changes in staffing or patient acuity over time in the NICU at GPHC that may have influenced the data. However, the impression among physicians and staff working in the NICU at GPHC is that the patient acuity has increased since 2015. There is also an initiative in the development stages to improve data gathering at GPHC and at the regional hospitals. Another limitation is that we only have data on neonates admitted to the NICU, so potential mortality of live born infants, and any changes over the study period occurring in labor and delivery, may have been missed. However, it is the practice that pediatrics is called for all high-risk deliveries and in most cases where advanced resuscitation efforts are needed (i.e., intubation, epinephrine, etc.) the patients are admitted to the NICU. Unfortunately, GPHC did not routinely use fetal monitoring during labor during the time of this study, so it is possible that there are a small number of stillbirths included in our data. Another limitation is the missing data in 2017 and 2018 due to lack of personnel to gather the data. Another potential limitation is the source of the NMR data for Guyana that was utilized for this study, although we did employ the UNICEF NMR dataset.

## Conclusions

We found that the implementation of a fully functioning Level III NICU at GPHC, where there had been no NICU previously, was associated with a sustained improvement in NICU survival rates in a resource limited setting. Given the observational nature of our study it is impossible to conclude causality, although the association is relatively strong and a more nuanced study design and data collection were not available during this time-period. In terms of the actual processes to get a fully functional Level III NICU implemented in this resource limited setting, it took longer than we had expected due to both anticipated and unanticipated barriers. The development and implementation of an entirely new NICU likely requires a collaborative approach individualized to the local circumstances, as well as resilience and agility on the part of leadership, staff, and physicians.

## Supporting information

S1 Data (XLSX)
